# Efficacy of comprehensive rehabilitation training for elderly patients with lower limb fractures after surgery

**DOI:** 10.12669/pjms.41.7.12125

**Published:** 2025-07

**Authors:** Yuxin Zhong, Hongchao Liu, Anqiang Li

**Affiliations:** 1 Yuxin Zhong, Emergency Trauma Center, Dongguan Chang’an Hospital, Dongguan, Guangdong Province 523850, P.R. China; 2 Hongchao Liu, Emergency Trauma Center, Dongguan Chang’an Hospital, Dongguan, Guangdong Province 523850, P.R. China; 3 Anqiang Li, Emergency Trauma Center, Dongguan Chang’an Hospital, Dongguan, Guangdong Province 523850, P.R. China

**Keywords:** Comprehensive rehabilitation training, Elderly patient, Lower limb fractures

## Abstract

**Objective:**

Exploring the efficacy of comprehensive rehabilitation training (CRT) for elderly patients with lower limb fractures (LLF) after surgery.

**Methods:**

This is a single center retrospective cohort study included clinical data of 120 elderly patients with LLF who underwent surgical treatment at Dongguan Chang’an Hospital from February 2021 to August 2023. Of them, 58 patients received routine rehabilitation training (control group) and 62 patients received CRT (observation group). Range of motion of lower limb joints, activities of daily living (ADLs), pain intensity, and incidence of complications were compared between the two groups.

**Results:**

After surgery, curvature, straightness, and degree of flexion and extension of the lower limb joints in both groups significantly increased, and was significantly greater in the observation group (*P*<0.05). Modified Barthel Index (MBI) scores of both groups significantly increased after surgery, and was significantly higher in the observation group (*P*<0.05). Post-surgery visual analog scale (VAS) scores of both groups significantly decreased, and were significantly lower in the observation group (*P*<0.05). The incidence of complications was significantly lower in the observation group than in the control group (*P*<0.05).

**Conclusions:**

Implementing CRT for elderly patients with LLF can increase the range of motion of the lower limb joints, improve ADLs, alleviate pain levels, and reduce the risk of complications.

## INTRODUCTION

Lower limb fractures (LLF) is prevalent in the elderly population, and often lead to a series of pathological injuries and ultimately result in functional impairment.^1^,^2^ LLF is associated with a very significant detrimental impact on the quality of life and physical and mental health of the elderly population.^3^–^5^

Surgery is considered an important measure for clinical treatment of LLF.^6^ However, long-term bed rest that is usually required after the surgery can easily lead to a lack of exercise and affect limb muscle strength in patients.^7^ Furthermore, it increases the risk of complications such as joint stiffness, muscle atrophy, and spasms, which can affect the effectiveness of functional rehabilitation.^6^,^7^ Therefore, effective postoperative rehabilitation training is crucial for elderly patients with LLF.^5^–^7^

Conventional rehabilitation training that is routinely used in clinical setting only focuses on guiding the functional training of the affected limb joints, thus lacking systematic and comprehensive approaches, resulting in poor overall effectiveness.^8^ Recent studies have showed that a more advanced method of postoperative rehabilitation, a comprehensive rehabilitation training (CRT) that provides a full rehabilitation guidance tailored to the patient’s physical condition, is highly effective in restoring joint mobility, reducing pain levels, and improving self-care abilities.^9^,^10^ This retrospective study aimed to analyzed clinical data of elderly patients with LLF to clarify the intervention effect of postoperative CRT.

## METHODS

In this single-center retrospective cohort study, clinical records of 120 elderly patients with LLF who underwent surgical treatment in Dongguan Chang’an Hospital from February 2021 to August 2023 were retrospectively selected. A total of 58 patients who received routine rehabilitation training were set as the control group, and 62 patients who received CRT were assigned to the observation group.

### Ethical approval:

The ethics committee of Dongguan Chang’an Hospital approved our study with the number LZ2025006; date: January 3, 2025.

### Inclusion criteria:

- Complete clinical data.
- LLF confirmed through computing tomography (CT), X-ray, or magnetic resonance imaging (MRI).
- Clear awareness and good compliance.
- Single site fracture.

### Exclusion criteria:

- Patients with comminuted fractures.
- Patients with open fractures.
- Patients with loss of normal walking and mobility before fracture.
- Patients with other bone and joint diseases/fractures.
- Patients with neurovascular injuries.
- Patients with mental illness and cognitive impairment.

### Routine rehabilitation training:

- Two hours after the surgery, patients were assisted in lying flat with the pillow removed, and centripetal massage was applied to the lower limb muscles. Six hours after the surgery, patients were assisted in taking a semi recumbent position, with affected limb placed in an outward position of 30º. Patients were demonstrated how to properly expel phlegm.
- Patients were assisted and guided to perform passive training such as ankle dorsiflexion and quadriceps isometric contraction exercises on the hospital bed 24 hours after the surgery, 3-5 times per day, 5-10 minutes per session.
- Around 24-48 hours after the surgery, patients were guided to perform passive exercises such as hip flexion and quadriceps contraction in bed, 3-5 times per day, 10-15 minutes per session.
- Three to seven days after the surgery, patients were guided to perform passive flexion and extension exercises such as ankle, knee, and hip using lower limb rehabilitation devices. Gradually, flexion and extension angle were increased according to the patient’s tolerance level. Exercises were performed 2-3 times per day, 30 minutes per time.
- One to two weeks post-surgery, patients were guided to perform bedside standing and sitting exercises, and encouraged to independently perform ankle, knee, hip flexion and extension exercises, as well as straight leg lifting exercises in bed, 2-3 times per day, 15-20 minutes per session.
- Two to four weeks after the procedure, patients were assisted and guided to perform hip muscle resistance exercises, 2-3 times per day, 10-15 minutes per session. Patients were guided to carry out weightless walking exercises based on their specific condition, twice a day, for 15-20 minutes per session.
- One to three months after the surgery, based on the X-ray examination results, patients were encouraged to engage in partial weight-bearing walking exercises. Weight was increased appropriately, according to the patient’s rehabilitation situation, and patients were gradually transitioned to walking exercises without crutches, twice a day, 20-30 minutes per session. The length of the intervention was eight weeks.

### CRT:

The CRT was carried out based on the routine rehabilitation training. Patients were guided to perform a set of exercises.

Proprioception exercise - multi angle hip joint repetitive exercises, flexing the hip at 30°, 45°, 60°, 90°, and 110° respectively, for 3-5 seconds each. Patients were instructed to train once with eyes closed and once with eyes open, two times a day, 15 minutes per session.

Proprioceptive neuromuscular exercises - patients were guided to lie flat, and stimulation such as compression and touch pressure to the affected limb, was provided. Patients were assisted in performing limb bending exercises, and guided to carry out antagonistic rhythmic stability exercises and isotonic combination exercises. Subsequently, patients were guided to take a prone position and perform exercises to relax and maintain the flexor muscles two times a day, 15 minutes per session. Balance exercise - patients were guided to stand against the wall on a balance pad. A Swiss ball was placed between the patient’s back and the wall, and patients were guided to spread their feet and shoulders equally wide, wrap their hands around their chest, slowly squat and hold for 10 seconds before gradually standing up. After resting for 10 seconds, the above steps were repeated, three times a day, 15 minutes per session.

Electric stimulation exercise - a medium frequency electrotherapy device was used to perform electrotherapy on the affected area. Electrotherapy parameters were based on the patient’s condition and tolerance. Exercise was carried out two times a day, 20 minutes per session.

Artificial Intelligence Rehabilitation Exercise - An intelligent knee joint continuous passive movement rehabilitation device was used, and the frequency set to 66 Hz, pulse count to 88, and interval time to two seconds. Swing angle of the rehabilitation period was set to 40° -110° -40°, and the training program was adjusted to passive flexion and extension. Exercise was performed 1 time a day, 30 minutes per session; MOTomed intelligent motion system was used to assist patients in sitting and performing lower limb pedaling and circular rotation exercises, twice a day for 20 minutes per session. Intervention lasted for eight weeks.

### Observation indicators:

- Lower limb joint range of motion, including flexion and extension curvature, straightness, and flexion and extension.
- Activities of daily living (ADLs)Daily living activity ability, evaluated based on the modified Barthel index (MBI) scale, including feeding, personal hygiene, bathing, dressing, chair-bed transfer, toileting, bladder continence, bowel continence, ambulation or wheelchair use, and stair climbingbowel and bowel control, going up and down stairs, bed and chair transfer, walking on flat ground, etc., with a total score of 100 points. Higher MBI score indicated better daily life activity ability.
- Pain intensity assessed using visual analogue scale (VAS). VAS has a total of 10 points, with lower score indicating lower pain severity.
- The incidence of complications, including traumatic arthritis, malunion, joint stiffness, and deep vein thrombosis (DVT) in the lower limbs.

### Statistical analysis:

All data analyses were conducted using SPSS 20.0 software (IBM Corp, Armonk, NY, USA) and PRISM 8.0 software (GraphPad, San Diego, USA). Quantitative data were represented by mean ± standard deviation, independent sample *t*-test was used for intergroup comparison, and paired *t*-test was used for intra group before and after comparison. Count data were shown as number of cases and analyzed using Chi square test. *P*<0.05 was considered statistically significant.

## RESULTS

A total of 120 patients (61 males and 59 females) met the eligibility criteria for this study. Age of the patients ranged from 60 to 83 years old, with an average of 69.53 ± 5.10 years. A total of 58 patients received routine rehabilitation training after the surgery, and 62 patients received CRT. There was no significant difference in the baseline data between the groups (*P*>0.05) (Table-I).

**Table-I:** Comparison of general information between two groups.

Item	Observation group (n=62)	Control group (n=58)	t/χ^2^	P
Gender				
Male	33 (53.23)	28 (48.28)	0.294	0.588
Female	29 (46.77)	30 (51.72)		
Age (year)	70.10±4.69	68.93±5.48	1.255	0.212
Fracture type			2.124	0.547
Femoral fracture	21 (33.87)	24 (41.38)		
Patellar fracture	17 (27.42)	18 (31.03)		
Tibial fracture	14 (22.58)	11 (18.97)		
Fibular Fracture	10 (13.13)	5 (8.62)		
Affected side				
Left side	30 (48.39)	30 (51.72)	0.133	0.715
Right side	32 (51.61)	28 (48.28)		
Cause of injury			1.524	0.677
Fall	28 (45.16)	32 (55.17)		
Traffic accident	18 (29.03)	14 (24.14)		
Heavy object injury	11 (17.74)	7 (12.07)		
Others	5 (8.06)	5 (8.62)		

**Fig.1:**
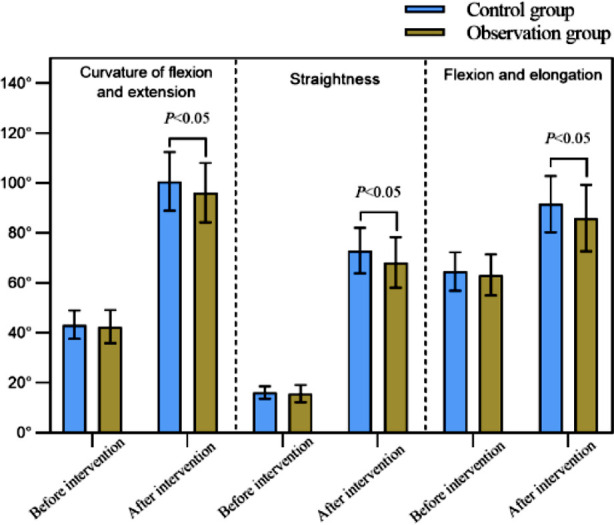
Comparison of flexion and extension curves, straightness and flexion and extension of lower limb joints between two groups.

Before the surgery, there was no significant difference in the curvature, straightness, and flexion and extension of the lower limb joints between the two groups (*P*>0.05); After the surgery, the curvature, straightness, and degree of flexion and extension of the lower limb joints in both groups significantly increased compared to before intervention, and was significantly larger in the observation group (*P*<0.05) (Fig.1).

**Fig.2:**
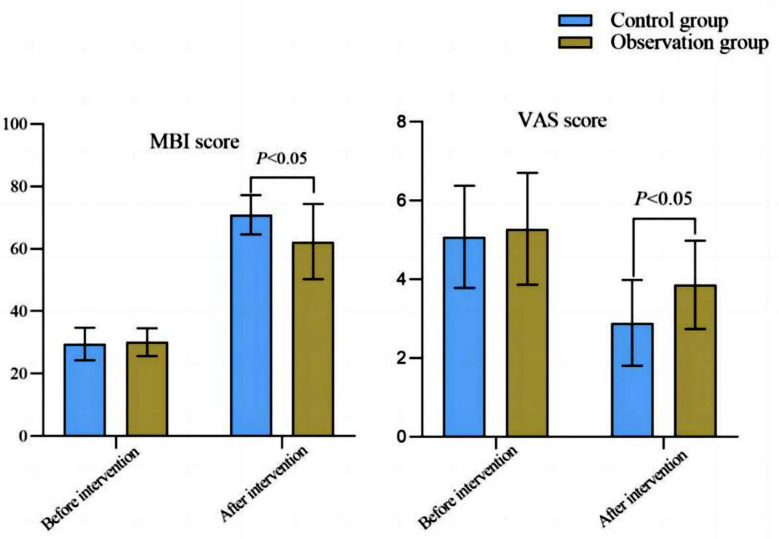
Comparison of MBI and VAS scores between two groups.

Before the surgery, there was no significant difference in MBI and VAS scores between the two groups (*P*>0.05). Post-surgery MBI scores of the two groups significantly increased compared to pre-surgery levels, and were significantly higher in the observation group compared to the control group (*P*<0.05). VAS score post-surgery significantly decreased compared to before the surgery, and were significantly lower in the observation group (*P*<0.05) (Fig.2). After the surgery, the complications in the observation group were significantly lower than those in the control group (*P*<0.05) (Table-II).

**Table-II:** Comparison of incidence rates of complications between two groups.

Group	n	Traumatic arthritis	Malunion	Joint stiffness	DVT	Total incidence rate
Observation group	62	1 (1.61)	1 (1.61)	1 (1.61)	0 (0.00)	3 (4.84)
Control group	58	3 (5.17)	2 (3.45)	4 (6.90)	1 (1.72)	10 (17.24)
*χ^2^*						4.772
*P*						0.029

## DISCUSSION

The results of this study indicate that CRT can more effectively improve physical function of elderly patients with LLF, alleviate pain levels, and facilitate increased joint mobility of the lower limbs, compared to the conventional rehabilitation training.

Our results showed that curvature, straightness, and flexion of the lower limb joints in the observation group were higher than those in the control group. Additionally, MBI scores in patients who received CRT were higher compared to patients who received conventional training, while VAS scores were lower (*P*<0.05). Our results are consistent with the study of Yashkov AV et al.^11^ that showed that CRT measures (including muscle electrical stimulation, muscle strengthening training, functional training, balance training, etc.) can effectively restore balance function, promote hip joint function recovery and improve overall quality of life of osteoporosis patients undergoing total hip replacement surgery. Related studies also indicate that since CRT has adopted multiple personalized intervention measures, it can provide targeted and systematic rehabilitation training programs that would maximize joint mobility, reduce pain, strengthening muscle, and improve limb function.^11^,^12^

Our study found that intelligent technology for CRT has positive significance in restoring joint mobility and function in patients with LLF. Picelli et al.^13^ also demonstrated that robot-assisted arm training is a feasible tool for postoperative rehabilitation of patients with upper limb fractures, which can help improve limb function and enhance their quality of life. Wu et al.^14^ confirmed that CRT measures can improve physical function and walking ability of patients with hip fractures, increase lower limb strength, and promote the recovery of daily living activities.

A study by Petya Kasnakova et al.^15^ confirmed that CRT measures can help shorten the process of functional recovery in patients with LLF. Additionally, they pointed out that CRT adopts various training measures to achieve different rehabilitation training objectives. For instance, joint traction can ensure effective reduction of fractures, ensuring the effectiveness of reduction and laying the foundation for fracture healing. Joint loosening training can prevent joint soft tissue adhesion and contraction, regulate blood circulation at the fracture site, reduce joint swelling, and accelerate joint soft tissue repair at the fracture site to improve joint mobility.^15^,^16^

Handoll et al.^17^ showed that the CRT intervention of LLF pays more attention to the principle of gradual progression. It establishes corresponding intervention measures based on the patient’s functional status and stage, from passive joint movement, active flexion and extension transition to weight-bearing exercises, proprioceptive exercises, systematically restoring coordination, balance, and stability of the fractured limb gradually and progressively.

The results of our study also showed that the incidence of complications in the observation group was lower than that in the control group (*P*<0.05). We may speculate that this effect comes from the nature of the CRT measures, which provide personalized and precise training from comprehensive aspects such as proprioception, proprioceptive neuromuscular, and balance based on the patient’s individual conditions, assisted by electrical stimulation and artificial intelligence.^18^ Muscle contraction and relaxation training improve blood circulation, enhances coordination of limb muscle groups, restores joint function, while active and passive knee flexion and extension exercises can stretch muscles and ligaments to a certain extent.^19^ This improved peripheral blood circulation allows to avoid complications such as joint adhesion and stiffness.

Routine rehabilitation training only focuses on guiding the functional training of the affected limb joints, lacks a systematic and comprehensive approach, and the overall effect is poor. Our results are consistent with the observations of Morville AL et al.,^20^ indicating that CRT measures can help reduce the incidence of postoperative complications in elderly patients with LLF. In summary, CRT has added electrical stimulation and intelligent assistive devices, which can improve blood circulation and cell metabolism. Moreover, the real-time feedback and progress display provided by intelligent devices enhance patients’ confidence in rehabilitation, resulting in higher postoperative rehabilitation benefits. The results of this study provide some new reference plans for postoperative rehabilitation training of elderly patients with LLF, which can use intelligent auxiliary equipment to improve rehabilitation effects.

### Limitations:

Firstly, this is a single-center retrospective study with a small sample size. Secondly, we found that the incidence of complications in the observation group was lower than that in the control group, which should be validated in future prospective studies with a large sample size to confirm that if the improvements were because of the effects of the CRT measures. Thirdly, surgical outcomes could have been influenced by human or technical factors, leading to variability in postoperative patient recovery. Fourthly, the impact of the two methods on the long-term functional recovery of the patients was not analyzed. Fifthly, the possible role of technology in the rehabilitation of orthopedic patients may affect rehabilitation outcomes, which should be further investigated in future research.

## CONCLUSION

CRT in elderly patients with LLF can increase the range of motion of the lower limb joints, promote the recovery of motor and balance functions, improve daily living activities, alleviate pain levels, and reduce the risk of complications. However, elderly patients have different individual conditions, with decreased physiological functions, underlying diseases, and poor compliance. So the rehabilitation plan needs to comprehensively consider these factors and develop personalized, safe, and effective rehabilitation plans.

### Authors’ contributions:

**YZ:** Study design, literature search, manuscript writing. **YZ, HL and AL:** Data collection, data analysis, interpretation, Manuscript revision and validation.

All authors have read and approved the final manuscript. They are also responsible for the integrity of the study.
